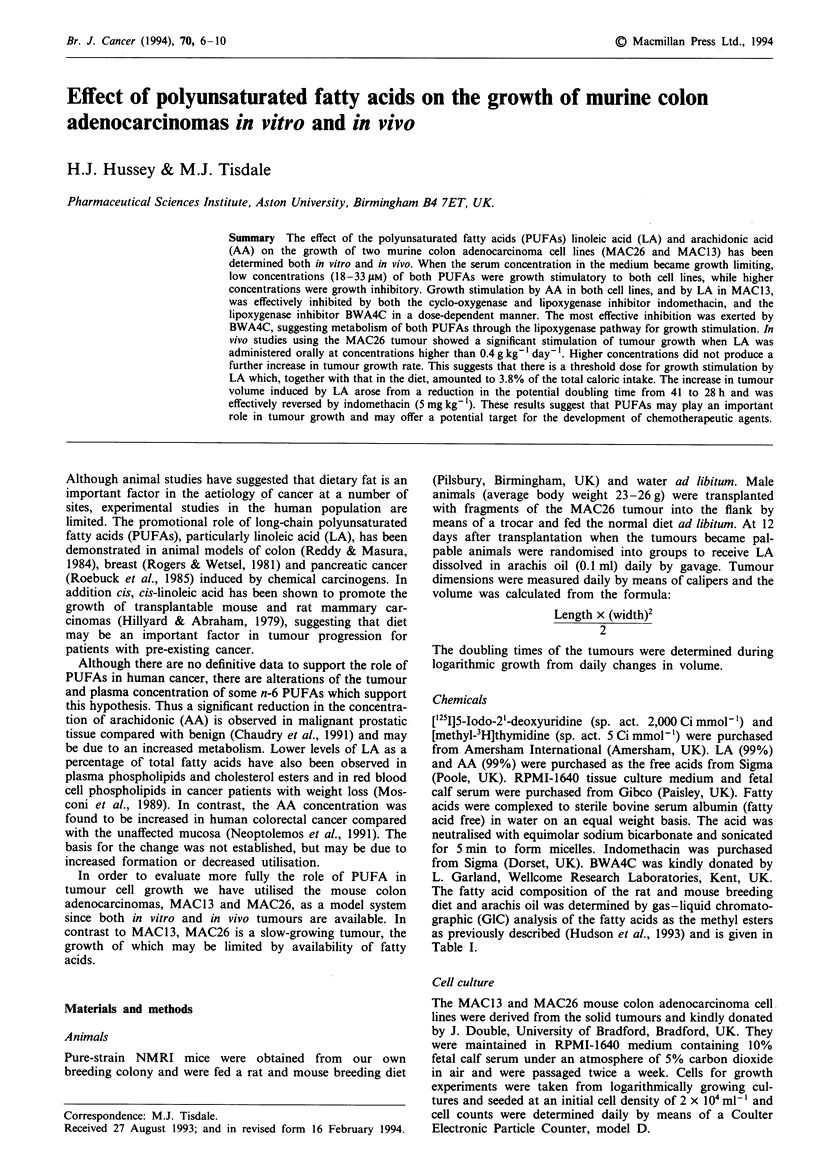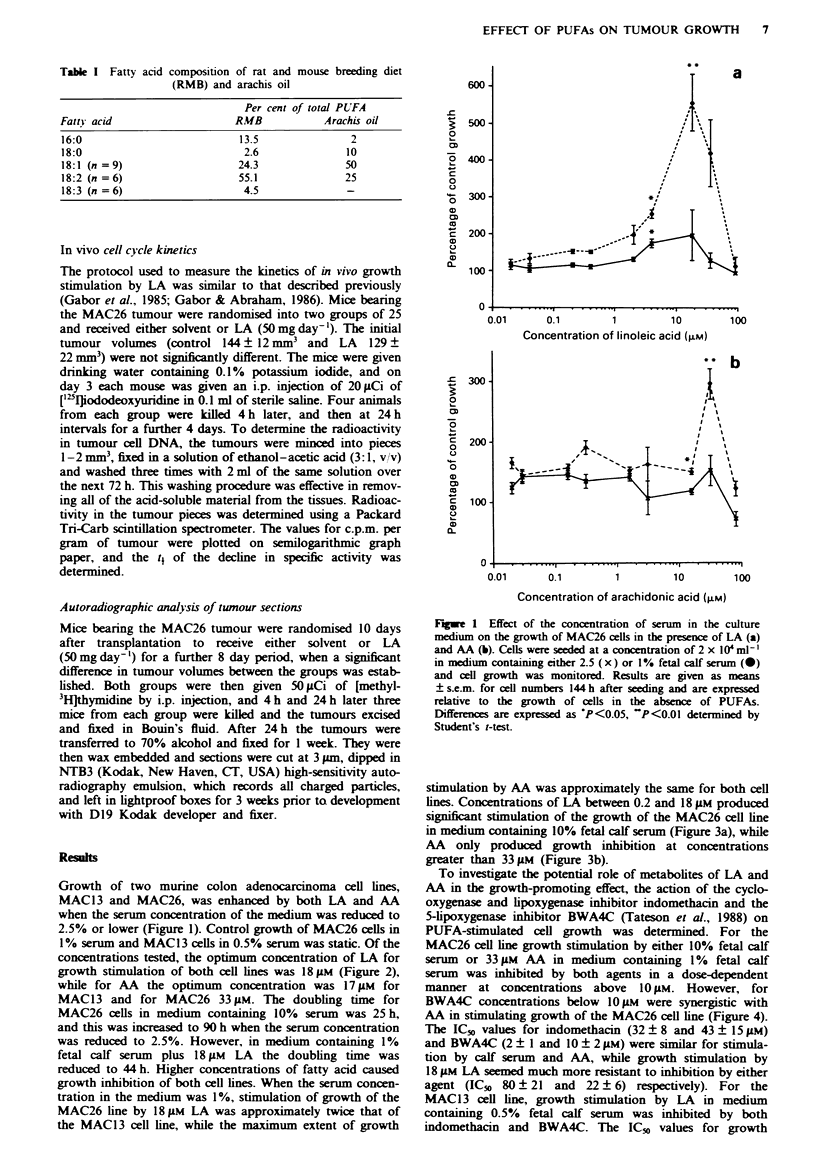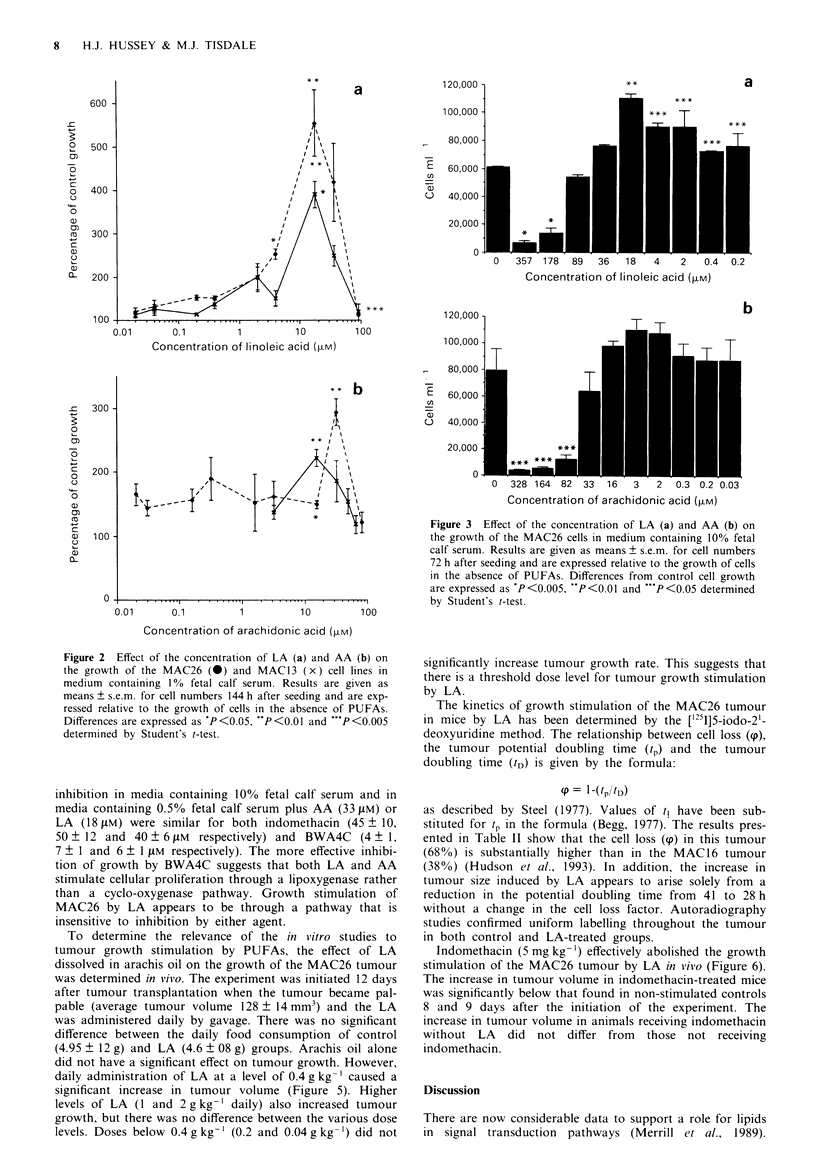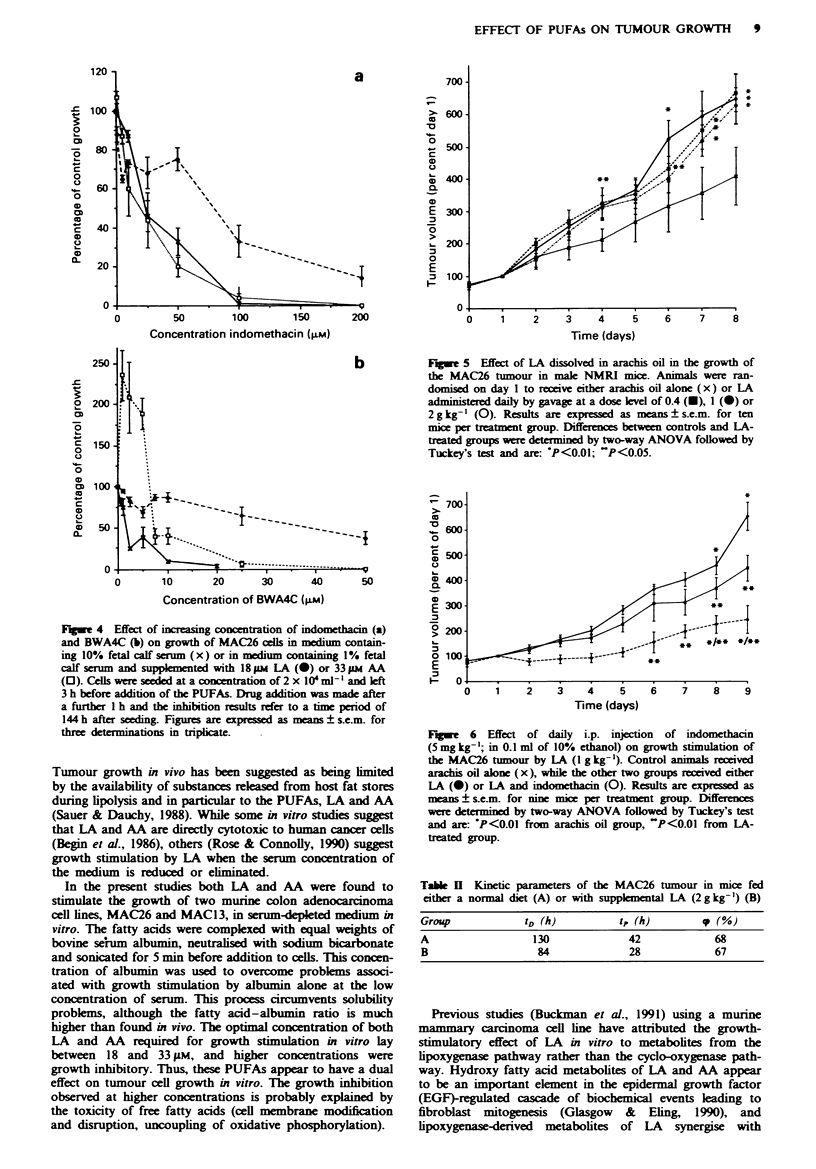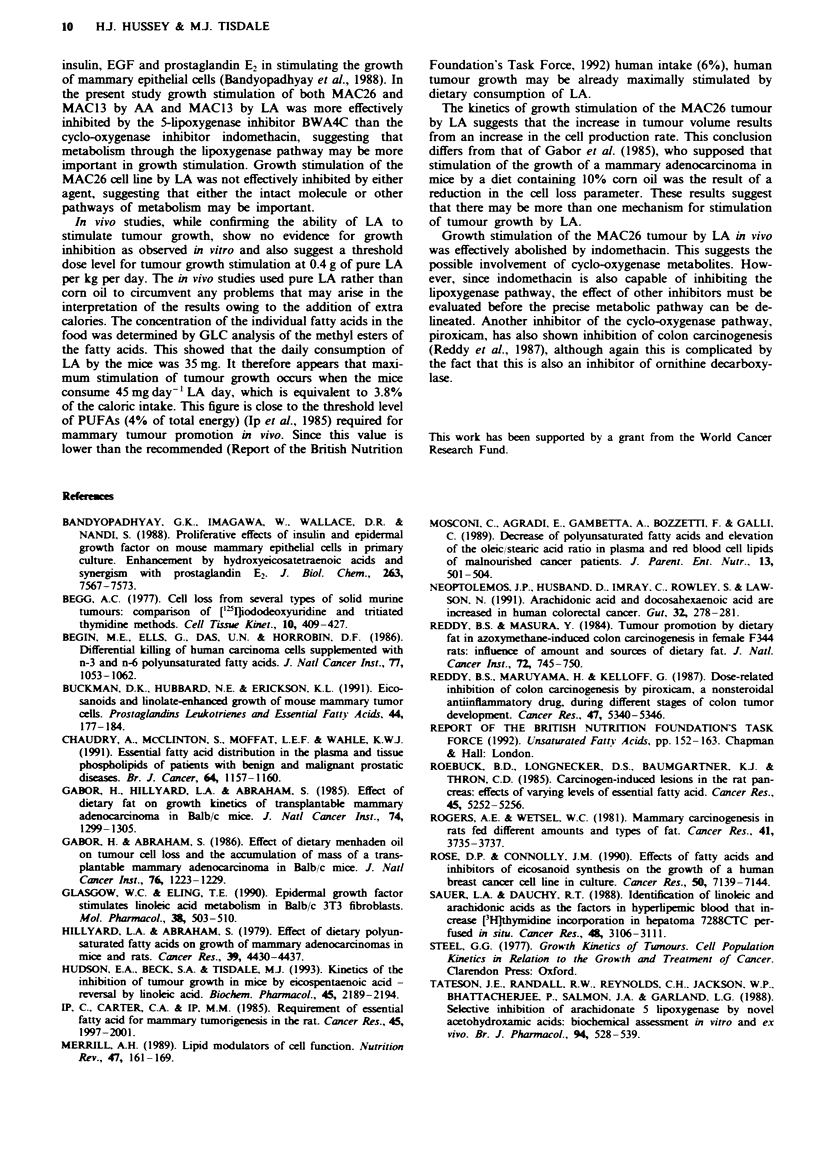# Effect of polyunsaturated fatty acids on the growth of murine colon adenocarcinomas in vitro and in vivo.

**DOI:** 10.1038/bjc.1994.241

**Published:** 1994-07

**Authors:** H. J. Hussey, M. J. Tisdale

**Affiliations:** Pharmaceutical Sciences Institute, Aston University, Birmingham, UK.

## Abstract

The effect of the polyunsaturated fatty acids (PUFAs) linoleic acid (LA) and arachidonic acid (AA) on the growth of two murine colon adenocarcinoma cell lines (MAC26 and MAC13) has been determined both in vitro and in vivo. When the serum concentrations in the medium became growth limiting, low concentrations (18-33 microM) of both PUFAs were growth stimulatory to both cell lines, while higher concentrations were growth inhibitory. Growth stimulation by AA in both cell lines, and by LA in MAC13, was effectively inhibited by both the cyclo-oxygenase and lipoxygenase inhibitor indomethacin, and the lipoxygenase inhibitor BWA4C in a dose-dependent manner. The most effective inhibition was exerted by BWA4C, suggesting metabolism of both PUFAs through the lipoxygenase pathway for growth stimulation. In vivo studies using the MAC26 tumour showed a significant stimulation of tumour growth when LA was administered orally at concentrations higher than 0.4 g kg-1 day-1. Higher concentrations did not produce a further increase in tumour growth rate. This suggests that there is a threshold dose for growth stimulation by LA which, together with that in the diet, amounted to 3.8% of the total caloric intake. The increase in tumour volume induced by LA arose from a reduction in the potential doubling time from 41 to 28 h and was effectively reversed by indomethacin (5 mg kg-1). These results suggest that PUFAs may play an important role in tumour growth and may offer a potential target for the development of chemotherapeutic agents.


Br. J. Cancer (1994), 70, 6–10 © Macmillan Press Ltd., 1994

### Effect of polyunsaturated fatty acids on the growth of murine colon adenocarcinomas *in vitro* and *in vivo*

H.J. Hussey & M.J. Tisdale

*Pharmaceutical Sciences Institute, Aston University, Birmingham B4 7ET, UK.*

**Summary** The effect of the polyunsaturated fatty acids (PUFAs) linoleic acid (LA) and arachidonic acid (AA) on the growth of two murine colon adenocarcinoma cell lines (MAC26 and MAC13) has been determined both *in vitro* and *in vivo*. When the serum concentration in the medium became growth limiting, low concentrations (18–33 μM) of both PUFAs were growth stimulatory to both cell lines, while higher concentrations were growth inhibitory. Growth stimulation by AA in both cell lines, and by LA in MAC13, was effectively inhibited by both the cyclo-oxygenase and lipoxygenase inhibitor indomethacin, and the lipoxygenase inhibitor BWA4C in a dose-dependent manner. The most effective inhibition was exerted by BWA4C, suggesting metabolism of both PUFAs through the lipoxygenase pathway for growth stimulation. *In vivo* studies using the MAC26 tumour showed a significant stimulation of tumour growth when LA was administered orally at concentrations higher than 0.4 g $kg^{-1}$ $day^{-1}$. Higher concentrations did not produce a further increase in tumour growth rate. This suggests that there is a threshold dose for growth stimulation by LA which, together with that in the diet, amounted to 3.8% of the total caloric intake. The increase in tumour volume induced by LA arose from a reduction in the potential doubling time from 41 to 28 h and was effectively reversed by indomethacin (5 mg $kg^{-1}$). These results suggest that PUFAs may play an important role in tumour growth and may offer a potential target for the development of chemotherapeutic agents.

Although animal studies have suggested that dietary fat is an important factor in the aetiology of cancer at a number of sites, experimental studies in the human population are limited. The promotional role of long-chain polyunsaturated fatty acids (PUFAs), particularly linoleic acid (LA), has been demonstrated in animal models of colon (Reddy & Masura, 1984), breast (Rogers & Wetsel, 1981) and pancreatic cancer (Roebuck *et al.*, 1985) induced by chemical carcinogens. In addition *cis*, *cis*-linoleic acid has been shown to promote the growth of transplantable mouse and rat mammary carcinomas (Hillyard & Abraham, 1979), suggesting that diet may be an important factor in tumour progression for patients with pre-existing cancer.

Although there are no definitive data to support the role of PUFAs in human cancer, there are alterations of the tumour and plasma concentration of some *n*-6 PUFAs which support this hypothesis. Thus a significant reduction in the concentration of arachidonic (AA) is observed in malignant prostatic tissue compared with benign (Chaudry *et al.*, 1991) and may be due to an increased metabolism. Lower levels of LA as a percentage of total fatty acids have also been observed in plasma phospholipids and cholesterol esters and in red blood cell phospholipids in cancer patients with weight loss (Mosconi *et al.*, 1989). In contrast, the AA concentration was found to be increased in human colorectal cancer compared with the unaffected mucosa (Neoptolemos *et al.*, 1991). The basis for the change was not established, but may be due to increased formation or decreased utilisation.

In order to evaluate more fully the role of PUFA in tumour cell growth we have utilised the mouse colon adenocarcinomas, MAC13 and MAC26, as a model system since both *in vitro* and *in vivo* tumours are available. In contrast to MAC13, MAC26 is a slow-growing tumour, the growth of which may be limited by availability of fatty acids.

### Materials and methods

### Animals

Pure-strain NMRI mice were obtained from our own breeding colony and were fed a rat and mouse breeding diet (Pilsbury, Birmingham, UK) and water *ad libitum*. Male animals (average body weight 23–26 g) were transplanted with fragments of the MAC26 tumour into the flank by means of a trocar and fed the normal diet *ad libitum*. At 12 days after transplantation when the tumours became palpable animals were randomised into groups to receive LA dissolved in arachis oil (0.1 ml) daily by gavage. Tumour dimensions were measured daily by means of calipers and the volume was calculated from the formula:

$$\frac{\text{Length} \times (\text{width})^2}{2}$$

The doubling times of the tumours were determined during logarithmic growth from daily changes in volume.

### Chemicals

[$^{125}$I]5-Iodo-2$^{1}$-deoxyuridine (sp. act. 2,000 Ci $mmol^{-1}$) and [methyl-$^{3}$H]thymidine (sp. act. 5 Ci $mmol^{-1}$) were purchased from Amersham International (Amersham, UK). LA (99%) and AA (99%) were purchased as the free acids from Sigma (Poole, UK). RPMI-1640 tissue culture medium and fetal calf serum were purchased from Gibco (Paisley, UK). Fatty acids were complexed to sterile bovine serum albumin (fatty acid free) in water on an equal weight basis. The acid was neutralised with equimolar sodium bicarbonate and sonicated for 5 min to form micelles. Indomethacin was purchased from Sigma (Dorset, UK). BWA4C was kindly donated by L. Garland, Wellcome Research Laboratories, Kent, UK. The fatty acid composition of the rat and mouse breeding diet and arachis oil was determined by gas–liquid chromatographic (GlC) analysis of the fatty acids as the methyl esters as previously described (Hudson *et al.*, 1993) and is given in Table I.

### Cell culture

The MAC13 and MAC26 mouse colon adenocarcinoma cell lines were derived from the solid tumours and kindly donated by J. Double, University of Bradford, Bradford, UK. They were maintained in RPMI-1640 medium containing 10% fetal calf serum under an atmosphere of 5% carbon dioxide in air and were passaged twice a week. Cells for growth experiments were taken from logarithmically growing cultures and seeded at an initial cell density of $2 \times 10^4$ $ml^{-1}$ and cell counts were determined daily by means of a Coulter Electronic Particle Counter, model D.

Correspondence: M.J. Tisdale.
Received 27 August 1993; and in revised form 16 February 1994.

**Table I** Fatty acid composition of rat and mouse breeding diet (RMB) and arachis oil

| | *Per cent of total PUFA* | |
|---|---|---|
| *Fatty acid* | *RMB* | *Arachis oil* |
| 16:0 | 13.5 | 2 |
| 18:0 | 2.6 | 10 |
| 18:1 ($n$ = 9) | 24.3 | 50 |
| 18:2 ($n$ = 6) | 55.1 | 25 |
| 18:3 ($n$ = 6) | 4.5 | – |

### In vivo *cell cycle kinetics*

The protocol used to measure the kinetics of *in vivo* growth stimulation by LA was similar to that described previously (Gabor *et al.*, 1985; Gabor & Abraham, 1986). Mice bearing the MAC26 tumour were randomised into two groups of 25 and received either solvent or LA (50 mg day$^{-1}$). The initial tumour volumes (control 144 ± 12 mm$^3$ and LA 129 ± 22 mm$^3$) were not significantly different. The mice were given drinking water containing 0.1% potassium iodide, and on day 3 each mouse was given an i.p. injection of 20 μCi of [$^{125}$I]iododeoxyuridine in 0.1 ml of sterile saline. Four animals from each group were killed 4 h later, and then at 24 h intervals for a further 4 days. To determine the radioactivity in tumour cell DNA, the tumours were minced into pieces 1–2 mm$^3$, fixed in a solution of ethanol–acetic acid (3:1, v/v) and washed three times with 2 ml of the same solution over the next 72 h. This washing procedure was effective in removing all of the acid-soluble material from the tissues. Radioactivity in the tumour pieces was determined using a Packard Tri-Carb scintillation spectrometer. The values for c.p.m. per gram of tumour were plotted on semilogarithmic graph paper, and the $t_{\frac{1}{2}}$ of the decline in specific activity was determined.

### *Autoradiographic analysis of tumour sections*

Mice bearing the MAC26 tumour were randomised 10 days after transplantation to receive either solvent or LA (50 mg day$^{-1}$) for a further 8 day period, when a significant difference in tumour volumes between the groups was established. Both groups were then given 50 μCi of [methyl-$^3$H]thymidine by i.p. injection, and 4 h and 24 h later three mice from each group were killed and the tumours excised and fixed in Bouin's fluid. After 24 h the tumours were transferred to 70% alcohol and fixed for 1 week. They were then wax embedded and sections were cut at 3 μm, dipped in NTB3 (Kodak, New Haven, CT, USA) high-sensitivity autoradiography emulsion, which records all charged particles, and left in lightproof boxes for 3 weeks prior to development with D19 Kodak developer and fixer.

### Results

Growth of two murine colon adenocarcinoma cell lines, MAC13 and MAC26, was enhanced by both LA and AA when the serum concentration of the medium was reduced to 2.5% or lower (Figure 1). Control growth of MAC26 cells in 1% serum and MAC13 cells in 0.5% serum was static. Of the concentrations tested, the optimum concentration of LA for growth stimulation of both cell lines was 18 μM (Figure 2), while for AA the optimum concentration was 17 μM for MAC13 and for MAC26 33 μM. The doubling time for MAC26 cells in medium containing 10% serum was 25 h, and this was increased to 90 h when the serum concentration was reduced to 2.5%. However, in medium containing 1% fetal calf serum plus 18 μM LA the doubling time was reduced to 44 h. Higher concentrations of fatty acid caused growth inhibition of both cell lines. When the serum concentration in the medium was 1%, stimulation of growth of the MAC26 line by 18 μM LA was approximately twice that of the MAC13 cell line, while the maximum extent of growth

**Figure 1** Effect of the concentration of serum in the culture medium on the growth of MAC26 cells in the presence of LA (**a**) and AA (**b**). Cells were seeded at a concentration of 2 × 10$^4$ ml$^{-1}$ in medium containing either 2.5 (×) or 1% fetal calf serum (●) and cell growth was monitored. Results are given as means ± s.e.m. for cell numbers 144 h after seeding and are expressed relative to the growth of cells in the absence of PUFAs. Differences are expressed as $^*P$<0.05, $^{**}P$<0.01 determined by Student's $t$-test.

stimulation by AA was approximately the same for both cell lines. Concentrations of LA between 0.2 and 18 μM produced significant stimulation of the growth of the MAC26 cell line in medium containing 10% fetal calf serum (Figure 3a), while AA only produced growth inhibition at concentrations greater than 33 μM (Figure 3b).

To investigate the potential role of metabolites of LA and AA in the growth-promoting effect, the action of the cyclooxygenase and lipoxygenase inhibitor indomethacin and the 5-lipoxygenase inhibitor BWA4C (Tateson *et al.*, 1988) on PUFA-stimulated cell growth was determined. For the MAC26 cell line growth stimulation by either 10% fetal calf serum or 33 μM AA in medium containing 1% fetal calf serum was inhibited by both agents in a dose-dependent manner at concentrations above 10 μM. However, for BWA4C concentrations below 10 μM were synergistic with AA in stimulating growth of the MAC26 cell line (Figure 4). The IC$_{50}$ values for indomethacin (32 ± 8 and 43 ± 15 μM) and BWA4C (2 ± 1 and 10 ± 2 μM) were similar for stimulation by calf serum and AA, while growth stimulation by 18 μM LA seemed much more resistant to inhibition by either agent (IC$_{50}$ 80 ± 21 and 22 ± 6) respectively). For the MAC13 cell line, growth stimulation by LA in medium containing 0.5% fetal calf serum was inhibited by both indomethacin and BWA4C. The IC$_{50}$ values for growth

**Figure 2** Effect of the concentration of LA (**a**) and AA (**b**) on the growth of the MAC26 (●) and MAC13 (×) cell lines in medium containing 1% fetal calf serum. Results are given as means ± s.e.m. for cell numbers 144 h after seeding and are expressed relative to the growth of cells in the absence of PUFAs. Differences are expressed as *$P<0.05$, **$P<0.01$ and ***$P<0.005$ determined by Student's *t*-test.

**Figure 3** Effect of the concentration of LA (**a**) and AA (**b**) on the growth of the MAC26 cells in medium containing 10% fetal calf serum. Results are given as means ± s.e.m. for cell numbers 72 h after seeding and are expressed relative to the growth of cells in the absence of PUFAs. Differences from control cell growth are expressed as *$P<0.005$, **$P<0.01$ and ***$P<0.05$ determined by Student's *t*-test.

inhibition in media containing 10% fetal calf serum and in media containing 0.5% fetal calf serum plus AA (33 μM) or LA (18 μM) were similar for both indomethacin (45 ± 10, 50 ± 12 and 40 ± 6 μM respectively) and BWA4C (4 ± 1, 7 ± 1 and 6 ± 1 μM respectively). The more effective inhibition of growth by BWA4C suggests that both LA and AA stimulate cellular proliferation through a lipoxygenase rather than a cyclo-oxygenase pathway. Growth stimulation of MAC26 by LA appears to be through a pathway that is insensitive to inhibition by either agent.

To determine the relevance of the *in vitro* studies to tumour growth stimulation by PUFAs, the effect of LA dissolved in arachis oil on the growth of the MAC26 tumour was determined *in vivo*. The experiment was initiated 12 days after tumour transplantation when the tumour became palpable (average tumour volume 128 ± 14 mm$^3$) and the LA was administered daily by gavage. There was no significant difference between the daily food consumption of control (4.95 ± 12 g) and LA (4.6 ± 08 g) groups. Arachis oil alone did not have a significant effect on tumour growth. However, daily administration of LA at a level of 0.4 g kg$^{-1}$ caused a significant increase in tumour volume (Figure 5). Higher levels of LA (1 and 2 g kg$^{-1}$ daily) also increased tumour growth, but there was no difference between the various dose levels. Doses below 0.4 g kg$^{-1}$ (0.2 and 0.04 g kg$^{-1}$) did not significantly increase tumour growth rate. This suggests that there is a threshold dose level for tumour growth stimulation by LA.

The kinetics of growth stimulation of the MAC26 tumour in mice by LA has been determined by the [$^{125}$I]5-iodo-2$^1$-deoxyuridine method. The relationship between cell loss ($\varphi$), the tumour potential doubling time ($t_p$) and the tumour doubling time ($t_D$) is given by the formula:

$$\varphi = 1\text{-}(t_p/t_D)$$

as described by Steel (1977). Values of $t_l$ have been substituted for $t_p$ in the formula (Begg, 1977). The results presented in Table II show that the cell loss ($\varphi$) in this tumour (68%) is substantially higher than in the MAC16 tumour (38%) (Hudson *et al.*, 1993). In addition, the increase in tumour size induced by LA appears to arise solely from a reduction in the potential doubling time from 41 to 28 h without a change in the cell loss factor. Autoradiography studies confirmed uniform labelling throughout the tumour in both control and LA-treated groups.

Indomethacin (5 mg kg$^{-1}$) effectively abolished the growth stimulation of the MAC26 tumour by LA *in vivo* (Figure 6). The increase in tumour volume in indomethacin-treated mice was significantly below that found in non-stimulated controls 8 and 9 days after the initiation of the experiment. The increase in tumour volume in animals receiving indomethacin without LA did not differ from those not receiving indomethacin.

### Discussion

There are now considerable data to support a role for lipids in signal transduction pathways (Merrill *et al.*, 1989).

**Figure 4** Effect of increasing concentration of indomethacin (**a**) and BWA4C (**b**) on growth of MAC26 cells in medium containing 10% fetal calf serum (×) or in medium containing 1% fetal calf serum and supplemented with 18 μM LA (●) or 33 μM AA (□). Cells were seeded at a concentration of $2 \times 10^4\ ml^{-1}$ and left 3 h before addition of the PUFAs. Drug addition was made after a further 1 h and the inhibition results refer to a time period of 144 h after seeding. Figures are expressed as means ± s.e.m. for three determinations in triplicate.

Tumour growth *in vivo* has been suggested as being limited by the availability of substances released from host fat stores during lipolysis and in particular to the PUFAs, LA and AA (Sauer & Dauchy, 1988). While some *in vitro* studies suggest that LA and AA are directly cytotoxic to human cancer cells (Begin *et al*., 1986), others (Rose & Connolly, 1990) suggest growth stimulation by LA when the serum concentration of the medium is reduced or eliminated.

In the present studies both LA and AA were found to stimulate the growth of two murine colon adenocarcinoma cell lines, MAC26 and MAC13, in serum-depleted medium *in vitro*. The fatty acids were complexed with equal weights of bovine serum albumin, neutralised with sodium bicarbonate and sonicated for 5 min before addition to cells. This concentration of albumin was used to overcome problems associated with growth stimulation by albumin alone at the low concentration of serum. This process circumvents solubility problems, although the fatty acid–albumin ratio is much higher than found *in vivo*. The optimal concentration of both LA and AA required for growth stimulation *in vitro* lay between 18 and 33 μM, and higher concentrations were growth inhibitory. Thus, these PUFAs appear to have a dual effect on tumour cell growth *in vitro*. The growth inhibition observed at higher concentrations is probably explained by the toxicity of free fatty acids (cell membrane modification and disruption, uncoupling of oxidative phosphorylation).

**Figure 5** Effect of LA dissolved in arachis oil in the growth of the MAC26 tumour in male NMRI mice. Animals were randomised on day 1 to receive either arachis oil alone (×) or LA administered daily by gavage at a dose level of 0.4 (■), 1 (●) or $2\ g\ kg^{-1}$ (O). Results are expressed as means ± s.e.m. for ten mice per treatment group. Differences between controls and LA-treated groups were determined by two-way ANOVA followed by Tuckey's test and are: $^*P<0.01$; $^{**}P<0.05$.

**Figure 6** Effect of daily i.p. injection of indomethacin ($5\ mg\ kg^{-1}$; in 0.1 ml of 10% ethanol) on growth stimulation of the MAC26 tumour by LA ($1\ g\ kg^{-1}$). Control animals received arachis oil alone (×), while the other two groups received either LA (●) or LA and indomethacin (O). Results are expressed as means ± s.e.m. for nine mice per treatment group. Differences were determined by two-way ANOVA followed by Tuckey's test and are: $^*P<0.01$ from arachis oil group, $^{**}P<0.01$ from LA-treated group.

**Table II** Kinetic parameters of the MAC26 tumour in mice fed either a normal diet (A) or with supplemental LA ($2\ g\ kg^{-1}$) (B)

| *Group* | $t_D$ (h) | $t_P$ (h) | $\varphi$ (%) |
|---|---|---|---|
| A | 130 | 42 | 68 |
| B | 84 | 28 | 67 |

Previous studies (Buckman *et al*., 1991) using a murine mammary carcinoma cell line have attributed the growth-stimulatory effect of LA *in vitro* to metabolites from the lipoxygenase pathway rather than the cyclo-oxygenase pathway. Hydroxy fatty acid metabolites of LA and AA appear to be an important element in the epidermal growth factor (EGF)-regulated cascade of biochemical events leading to fibroblast mitogenesis (Glasgow & Eling, 1990), and lipoxygenase-derived metabolites of LA synergise with insulin, EGF and prostaglandin $E_2$ in stimulating the growth of mammary epithelial cells (Bandyopadhyay *et al.*, 1988). In the present study growth stimulation of both MAC26 and MAC13 by AA and MAC13 by LA was more effectively inhibited by the 5-lipoxygenase inhibitor BWA4C than the cyclo-oxygenase inhibitor indomethacin, suggesting that metabolism through the lipoxygenase pathway may be more important in growth stimulation. Growth stimulation of the MAC26 cell line by LA was not effectively inhibited by either agent, suggesting that either the intact molecule or other pathways of metabolism may be important.

*In vivo* studies, while confirming the ability of LA to stimulate tumour growth, show no evidence for growth inhibition as observed *in vitro* and also suggest a threshold dose level for tumour growth stimulation at 0.4 g of pure LA per kg per day. The *in vivo* studies used pure LA rather than corn oil to circumvent any problems that may arise in the interpretation of the results owing to the addition of extra calories. The concentration of the individual fatty acids in the food was determined by GLC analysis of the methyl esters of the fatty acids. This showed that the daily consumption of LA by the mice was 35 mg. It therefore appears that maximum stimulation of tumour growth occurs when the mice consume 45 mg day$^{-1}$ LA day, which is equivalent to 3.8% of the caloric intake. This figure is close to the threshold level of PUFAs (4% of total energy) (Ip *et al.*, 1985) required for mammary tumour promotion *in vivo*. Since this value is lower than the recommended (Report of the British Nutrition Foundation's Task Force, 1992) human intake (6%), human tumour growth may be already maximally stimulated by dietary consumption of LA.

The kinetics of growth stimulation of the MAC26 tumour by LA suggests that the increase in tumour volume results from an increase in the cell production rate. This conclusion differs from that of Gabor *et al.* (1985), who supposed that stimulation of the growth of a mammary adenocarcinoma in mice by a diet containing 10% corn oil was the result of a reduction in the cell loss parameter. These results suggest that there may be more than one mechanism for stimulation of tumour growth by LA.

Growth stimulation of the MAC26 tumour by LA *in vivo* was effectively abolished by indomethacin. This suggests the possible involvement of cyclo-oxygenase metabolites. However, since indomethacin is also capable of inhibiting the lipoxygenase pathway, the effect of other inhibitors must be evaluated before the precise metabolic pathway can be delineated. Another inhibitor of the cyclo-oxygenase pathway, piroxicam, has also shown inhibition of colon carcinogenesis (Reddy *et al.*, 1987), although again this is complicated by the fact that this is also an inhibitor of ornithine decarboxylase.

This work has been supported by a grant from the World Cancer Research Fund.

**References**

BANDYOPADHYAY, G.K., IMAGAWA, W., WALLACE, D.R. & NANDI, S. (1988). Proliferative effects of insulin and epidermal growth factor on mouse mammary epithelial cells in primary culture. Enhancement by hydroxyeicosatetraenoic acids and synergism with prostaglandin $E_2$. *J. Biol. Chem.*, **263**, 7567-7573.

BEGG, A.C. (1977). Cell loss from several types of solid murine tumours: comparison of [$^{125}$I]iododeoxyuridine and tritiated thymidine methods. *Cell Tissue Kinet.*, **10**, 409-427.

BEGIN, M.E., ELLS, G., DAS, U.N. & HORROBIN, D.F. (1986). Differential killing of human carcinoma cells supplemented with n-3 and n-6 polyunsaturated fatty acids. *J. Natl Cancer Inst.*, **77**, 1053-1062.

BUCKMAN, D.K., HUBBARD, N.E. & ERICKSON, K.L. (1991). Eicosanoids and linolate-enhanced growth of mouse mammary tumor cells. *Prostaglandins Leukotrienes and Essential Fatty Acids*, **44**, 177-184.

CHAUDRY, A., McCLINTON, S., MOFFAT, L.E.F. & WAHLE, K.W.J. (1991). Essential fatty acid distribution in the plasma and tissue phospholipids of patients with benign and malignant prostatic diseases. *Br. J. Cancer*, **64**, 1157-1160.

GABOR, H., HILLYARD, L.A. & ABRAHAM, S. (1985). Effect of dietary fat on growth kinetics of transplantable mammary adenocarcinoma in Balb/c mice. *J. Natl Cancer Inst.*, **74**, 1299-1305.

GABOR, H. & ABRAHAM, S. (1986). Effect of dietary menhaden oil on tumour cell loss and the accumulation of mass of a transplantable mammary adenocarcinoma in Balb/c mice. *J. Natl Cancer Inst.*, **76**, 1223-1229.

GLASGOW, W.C. & ELING, T.E. (1990). Epidermal growth factor stimulates linoleic acid metabolism in Balb/c 3T3 fibroblasts. *Mol. Pharmacol.*, **38**, 503-510.

HILLYARD, L.A. & ABRAHAM, S. (1979). Effect of dietary polyunsaturated fatty acids on growth of mammary adenocarcinomas in mice and rats. *Cancer Res.*, **39**, 4430-4437.

HUDSON, E.A., BECK, S.A. & TISDALE, M.J. (1993). Kinetics of the inhibition of tumour growth in mice by eicospentaenoic acid - reversal by linoleic acid. *Biochem. Pharmacol.*, **45**, 2189-2194.

IP, C., CARTER, C.A. & IP, M.M. (1985). Requirement of essential fatty acid for mammary tumorigenesis in the rat. *Cancer Res.*, **45**, 1997-2001.

MERRILL, A.H. (1989). Lipid modulators of cell function. *Nutrition Rev.*, **47**, 161-169.

MOSCONI, C., AGRADI, E., GAMBETTA, A., BOZZETTI, F. & GALLI, C. (1989). Decrease of polyunsaturated fatty acids and elevation of the oleic/stearic acid ratio in plasma and red blood cell lipids of malnourished cancer patients. *J. Parent. Ent. Nutr.*, **13**, 501-504.

NEOPTOLEMOS, J.P., HUSBAND, D., IMRAY, C., ROWLEY, S. & LAWSON, N. (1991). Arachidonic acid and docosahexaenoic acid are increased in human colorectal cancer. *Gut*, **32**, 278-281.

REDDY, B.S. & MASURA, Y. (1984). Tumour promotion by dietary fat in azoxymethane-induced colon carcinogenesis in female F344 rats: influence of amount and sources of dietary fat. *J. Natl. Cancer Inst.*, **72**, 745-750.

REDDY, B.S., MARUYAMA, H. & KELLOFF, G. (1987). Dose-related inhibition of colon carcinogenesis by piroxicam, a nonsteroidal antiinflammatory drug, during different stages of colon tumor development. *Cancer Res.*, **47**, 5340-5346.

REPORT OF THE BRITISH NUTRITION FOUNDATION'S TASK FORCE (1992). *Unsaturated Fatty Acids*, pp. 152-163. Chapman & Hall: London.

ROEBUCK, B.D., LONGNECKER, D.S., BAUMGARTNER, K.J. & THRON, C.D. (1985). Carcinogen-induced lesions in the rat pancreas: effects of varying levels of essential fatty acid. *Cancer Res.*, **45**, 5252-5256.

ROGERS, A.E. & WETSEL, W.C. (1981). Mammary carcinogenesis in rats fed different amounts and types of fat. *Cancer Res.*, **41**, 3735-3737.

ROSE, D.P. & CONNOLLY, J.M. (1990). Effects of fatty acids and inhibitors of eicosanoid synthesis on the growth of a human breast cancer cell line in culture. *Cancer Res.*, **50**, 7139-7144.

SAUER, L.A. & DAUCHY, R.T. (1988). Identification of linoleic and arachidonic acids as the factors in hyperlipemic blood that increase [$^3$H]thymidine incorporation in hepatoma 7288CTC perfused *in situ*. *Cancer Res.*, **48**, 3106-3111.

STEEL, G.G. (1977). *Growth Kinetics of Tumours. Cell Population Kinetics in Relation to the Growth and Treatment of Cancer.* Clarendon Press: Oxford.

TATESON, J.E., RANDALL, R.W., REYNOLDS, C.H., JACKSON, W.P., BHATTACHERJEE, P., SALMON, J.A. & GARLAND, L.G. (1988). Selective inhibition of arachidonate 5 lipoxygenase by novel acetohydroxamic acids: biochemical assessment *in vitro* and *ex vivo*. *Br. J. Pharmacol.*, **94**, 528-539.